# Parental meta-emotion philosophy and children’s classroom emotional regulation: the moderating role of teacher–student interaction quality

**DOI:** 10.3389/fpsyg.2026.1837821

**Published:** 2026-09-10

**Authors:** Na Zhao, Zhiyu Yao

**Affiliations:** 1 School of Preschool Education, Qingdao Preschool Education College, Qingdao, Shandong, China; 2 School of Journalism and Communication, Xinxiang University, Xinxiang, Henan, China

**Keywords:** bioecological model, classroom emotional regulation, emotion coaching, emotion dismissing, multilevel analysis, parental meta-emotion philosophy, teacher–student interaction

## Abstract

Whether children can manage affect inside the classroom now sits at the center of debates about school readiness, yet the family belief systems that shape this capacity are rarely examined within instructional settings. We asked how parental meta-emotion philosophy relates to change in two facets of children’s classroom emotional regulation—adaptive regulation and lability/negativity—across one academic year, and whether teacher–student interaction quality conditions those associations. A two-wave panel design with a 12-month interval followed 289 child–parent dyads (children aged 6–8 years) drawn from six public elementary schools in an eastern-Chinese metropolitan area. Parents completed the Chinese adaptation of the Maternal Emotional Style Questionnaire at baseline; trained observers coded children’s classroom emotional regulation at both waves; and teacher–student interaction quality was rated with the Classroom Assessment Scoring System, concurrently with the second wave of the outcome. Multilevel regression—children nested in classrooms—showed that emotion coaching at T1 forecast higher T2 adaptive regulation ( β = 0.17, *p* = 0.002) and, more modestly, lower lability/negativity ( β = − 0.11, *p* = 0.041), whereas emotion dismissing forecast lower adaptive regulation ( β = − 0.13, *p* = 0.018) and higher lability/negativity ( β = 0.15, *p* = 0.009), with baseline regulation, demographics, and classroom clustering controlled throughout. Teacher emotional support conditioned the family-level pathways on both facets: coaching effects were amplified where emotional support was high, whereas dismissing effects were partially buffered under the same condition. Classroom organization and instructional support moderated neither pathway. These findings extend meta-emotion theory into the instructional domain and offer evidence, in a Chinese context where coaching and dismissing beliefs may co-exist, for bioecological mesosystem interdependence between family and school microsystems.

## 1 Introduction

Emotion regulation inside the classroom sits at a curious intersection: it is neither a purely academic skill nor a purely socioemotional one, yet it conditions how well children take up almost everything else schools try to teach (Morris et al., 2007). A first-grader who holds on to frustration when a maths problem refuses to yield, or who scales back excitement long enough to hear a classmate’s turn, is not merely well-behaved; she is keeping the doors open to instruction (Blair and Raver, 2015). Where such regulation falters, teachers see the downstream signs—disengagement, peer conflict, thinning academic momentum—that longitudinal work has long linked to poor early self-regulation (Eisenberg et al., 2010). The developmental antecedents of this classroom-specific capacity, however, remain thinly mapped. Most attention has fallen on families as socialization contexts in the abstract, and far less on the belief systems that organize how parents respond to their children’s affect, or on how those beliefs meet what children encounter once the school door closes behind them.

Parental meta-emotion philosophy, introduced by Gottman et al. (1996), denotes the organized set of feelings and cognitions that parents hold about emotion itself—their own emotions and their children’s. Two broad orientations have received the greatest attention. Parents with an emotion-coaching orientation treat negative affect as an opportunity for teaching: they label emotions, validate their legitimacy, and scaffold coping (Gottman et al., 1997). Parents with an emotion-dismissing orientation, in contrast, perceive negative emotion as harmful or trivial and respond by minimizing, redirecting, or ignoring it (Lunkenheimer et al., 2007). Longitudinal work in Western samples ties coaching to superior vagal regulation, empathy, and adaptive peer interactions (Shortt et al., 2010), while cross-cultural studies caution that the same behaviors carry different meanings across cultural contexts (Chan et al., 2009). In Chinese families the picture is not simply attenuated but qualitatively different: coaching remains protective against internalizing symptoms (Han et al., 2015), yet coaching and dismissing endorsements can co-occur within the same parent, reflecting restraint-oriented socialization goals that Western taxonomies do not capture cleanly (Fiorilli et al., 2015; Wang et al., 2019; Raval, 2023). This co-existence, we will argue, matters for how meta-emotion should be modeled rather than only how it should be interpreted.

A second gap concerns the ecological context in which regulatory skills are enacted. Most measurement to date has relied on parent report or laboratory paradigms whose demands are only distantly related to what children navigate during instruction (Adrian et al., 2011). The classroom imposes its own constraints—externally structured time, authority relations with teachers, emotional challenges embedded in academic content—and these constraints call for regulatory strategies whose fit with home training is not automatic (Raver, 2004). Recent scholarship has therefore urged researchers to study classroom-situated regulation directly rather than in the aggregate (Eisenberg et al., 2010). That call has been only partially answered; empirical work linking family belief systems to in-class affective management is still scarce.

Whether the school context can compensate for what home does not provide, or amplify what home already offers, is a further empirical question. Teacher–student interaction quality—indexed by emotional support, classroom organization, and instructional support in Pianta’s Classroom Assessment Scoring System (Pianta et al., 2008)—has emerged as a robust predictor of children’s socioemotional adjustment on its own (Sabol and Pianta, 2012). From a bioecological standpoint, the mesosystem link between family and school microsystems should shape outcomes in ways that neither microsystem alone can explain (Bronfenbrenner and Morris, 2006). Warm, responsive teachers may offer alternative co-regulatory experiences that partially offset limited coaching at home (Mortensen and Barnett, 2018), or may reinforce the affective scripts that coaching parents have already installed. The differential susceptibility perspective adds a further nuance: children who are most sensitive to context should show the largest gains and the largest deficits, so the joint configuration of home and school ought to matter more than either component in isolation (Belsky and Pluess, 2009). Direct tests of teacher–student interaction as a moderator of the meta-emotion–classroom regulation link, however, remain scarce in the literature.

The present study addresses these gaps by modeling parental meta-emotion philosophy prospectively as a correlate of children’s classroom emotional regulation, with teacher–student interaction quality treated as a moderator whose measurement, we note upfront, is concurrent with the outcome and therefore not licensed to support causal claims. We followed a sample of primary-school-age children over 12 months and paired parental self-report with independent behavioral observation, a combination that limits common-method bias (Rogosa, 1980). Two questions organize the design. Does what parents believe about emotion at baseline forecast how children manage affect in class a year later, once prior regulation is taken into account? And does the affective quality of the classroom those children then inhabit condition that link? Because dual endorsement of coaching and dismissing is well documented in Chinese families (Fiorilli et al., 2015; Wang et al., 2019), the two orientations enter the analysis as separate continuous dimensions rather than as a categorical typology, and both facets of classroom regulation—adaptive regulation and lability/negativity—are carried through as outcomes. The specific predictions that follow from this framing are set out at the close of the literature review, once the constructs they involve have been developed.

This design speaks to several audiences. Theoretically, it extends meta-emotion accounts beyond global socioemotional outcomes into the instructional ecology, thereby bridging family and educational psychology (Morris et al., 2017), and it puts a testable operationalization on bioecological claims about mesosystem interdependence (Bronfenbrenner and Morris, 2006). Practically, the findings may inform parent education, by clarifying which belief patterns carry forward into school life, and teacher professional development, by locating the conditions under which family-origin regulatory tendencies find or lose their footing.

## 2 Theoretical foundations and literature review

### 2.1 Conceptual definition and typology of parental meta-emotion philosophy

Parental meta-emotion philosophy refers to the organized constellation of feelings, thoughts, and metaphors that parents hold about their own emotional experiences and those of their children (Gottman et al., 1996). Gottman and colleagues introduced the construct to capture not the discrete regulatory acts parents perform but the attitudinal scaffolding that shapes those acts (Katz et al., 2012). It thus operates at a meta-level: what parents believe emotion to be for, rather than what they do about it in the moment.

Four orientations are conventionally distinguished. Emotion-coaching parents treat children’s negative affect as normal and pedagogically useful, labeling feelings and using emotional episodes as teachable moments (Gottman et al., 1997). Emotion-dismissing parents view distress as damaging or trivial and respond by minimizing or redirecting expression (Lunkenheimer et al., 2007). A third, emotion-dysregulated orientation describes parents overwhelmed by their children’s affect, often because they lack confidence in managing their own (Katz and Windecker-Nelson, 2004). A fourth, emotion-laissez-faire orientation, captures parents who accept feelings but provide little guidance on behavioral boundaries or coping (Katz et al., 2012). A useful conceptual refinement, important for our analysis, is that emotion coaching itself is not internally uniform: an affect-acceptance sub-component (recognizing and validating feelings) coexists with an affect-shaping sub-component (teaching appropriate expression, sometimes involving restraint or control). The two can pull in opposing directions, especially for outcomes such as lability, and we return to this distinction when we interpret the observational measure below.

Measurement has evolved considerably since the original semi-structured meta-emotion interview (Gottman et al., 1996). Self-report questionnaires such as the Maternal Emotional Style Questionnaire (MESQ) enable larger samples, though critics note that abbreviated instruments may lose the nuance captured by interview (Lagacé-Séguin and Coplan, 2005). More recent adaptations complement self-report with observational coding of parent–child emotion dialogues (Baker et al., 2011). Neither approach is intrinsically superior; the trade-off is between ecological granularity and administrative practicality, and any given design must choose in light of sample size, cultural context, and the specific claims to be defended.

Cross-cultural work has complicated the picture in ways that bear directly on how meta-emotion should be modeled in Chinese samples. Emotion dismissal, in collectivist societies, does not necessarily carry the maladaptive connotations documented in Western data; some East Asian parents endorse restraint-oriented goals that overlap with dismissing attitudes but serve culturally adaptive functions (Tao et al., 2010; Chen and French, 2008). Empirically, Fiorilli et al. (2015) found that Hong Kong Chinese mothers scored higher than Italian mothers on both coaching and dismissing subscales of the MRCES, a pattern that runs against the assumption that the two orientations are mutually exclusive. Person-centered analyses reinforce the point: Wang and colleagues identified four latent profiles among Chinese fathers of adolescents—supportive, balanced, disengaged, and harsh—and Trevethan and colleagues extracted an emotion-disengaged profile distinct from a coaching profile in Chinese and Indian mothers (Wang et al., 2019; Trevethan et al., 2021). Raval (2023) recent synthesis has argued that pushing Global-North taxonomies onto Asian samples without such recalibration obscures more than it reveals. Two practical implications follow. First, culturally sensitive measurement is essential; second, and less commonly acknowledged, coaching and dismissing should be modeled as separate continuous dimensions rather than collapsed into a single categorical typology, because empirically they do co-vary in Chinese families in ways that dichotomies obscure. The MESQ Chinese adaptation used here yields these two subscales and has been applied successfully with Chinese caregivers in prior intervention and correlational work (Qiu and Shum, 2022).

### 2.2 Developmental features and influencing factors of children’s classroom emotional regulation

Classroom emotional regulation, as we operationalize it here, refers to a child’s ability to monitor, evaluate, and modify affective arousal in ways that sustain task engagement and social functioning within instructional settings (Thompson, 1994). The definition deliberately narrows the broader emotion-regulation construct to behaviors observable during structured learning episodes: managing frustration when a task resists solution, containing excitement during collaborative activities, waiting one’s turn to speak, and recovering composure after peer conflict or teacher correction. These are not merely convenient examples but the exact behaviors our observational instrument codes, and their context specificity distinguishes them from regulation as observed at bedtime, at dinner, or on the playground.

Two facets follow from this definition, and both are carried through the paper. Adaptive regulation denotes the constructive side of the construct—flexible recovery, situationally appropriate expression, sustained engagement under affective load. Lability/negativity denotes the dysregulated side—abrupt mood shifts, slow recovery from disappointment, and a negative affective tone that intrudes on classroom tasks. The two are inversely related but far from redundant, and they matter for partly different reasons: lability in particular has been tied to weaker classroom productivity and to poorer early achievement (Graziano et al., 2007). Keeping both facets in view, rather than reporting only the adaptive one, is what holds the construct as defined and the construct as measured in step with one another.

Developmentally, this capacity follows a broadly staged progression. Preschoolers depend heavily on adult co-regulation, whereas by middle childhood most can deploy internalized strategies such as cognitive reappraisal and selective attention shifting with growing autonomy (Calkins and Leerkes, 2011). Entry into formal schooling marks a critical juncture: classroom demands escalate sharply while the prefrontal circuitry that supports effortful control is still maturing (Zelazo and Carlson, 2012). Early primary years thus represent both a window of vulnerability and a sensitive period during which environmental supports can leave lasting marks.

Two broad classes of influence shape this trajectory. At the family level, parental emotion socialization stands out. Parents who label feelings, validate distress, and model adaptive coping equip children with a repertoire of regulatory strategies that transfers into instructional contexts (Shortt et al., 2010). The mechanism is not merely behavioral: through repeated coaching interactions, children internalize emotion knowledge—the capacity to identify and anticipate affective states—which in turn supports autonomous regulation when parents are absent (Denham et al., 2015). Dismissive or punitive responses, conversely, have been tied to reliance on suppression or avoidance, strategies that falter under the sustained demands of classroom participation (Eisenberg et al., 1998). At the school level, structural and relational features of the learning environment matter as well. Classroom climate, peer relations, and—most pertinently—teacher–student interaction constitute a second ecology of socialization (Hamre and Pianta, 2005; Sankalaite et al., 2021), to which we now turn.

### 2.3 Theoretical framework and moderating mechanisms of teacher–student interaction

Two theoretical traditions frame teacher–student interaction as a developmental influence. Bronfenbrenner’s bioecological model places the classroom as a microsystem whose proximal processes—daily, reciprocal exchanges between teacher and child—exert cumulative effects on socioemotional growth (Bronfenbrenner and Morris, 2006). Attachment theory adds a relational lens: teachers can act as secondary attachment figures whose responsiveness shapes children’s felt security and, by extension, their willingness to engage with emotionally challenging academic material (Verschueren and Koomen, 2012; Sabol and Pianta, 2012). Read together, the two frameworks make classroom relational quality a working ingredient of regulatory development rather than a passive backdrop.

The Classroom Assessment Scoring System (CLASS) operationalises this quality along three domains (Pianta et al., 2008). Emotional support captures the warmth, sensitivity, and regard for student perspectives that a teacher conveys. Classroom organization refers to behavioral management, productivity, and the provision of predictable routines. Instructional support encompasses the cognitive scaffolding and feedback loops that deepen conceptual understanding. All three bear on children’s affective experience, yet emotional support has shown the most consistent associations with regulatory outcomes, presumably because it directly models and reinforces adaptive affect management (Mashburn et al., 2008). We nevertheless retain all three domains throughout the analyses, partly because instructional support has been argued to matter for engagement-dependent regulation, and partly because a selective report would leave the reader wondering whether an omitted term hides an unexpected pattern.

A growing body of evidence suggests that teacher–student interaction moderates the link between family risk and child adjustment. High-quality emotional support has attenuated the negative developmental consequences of insecure parent–child attachment (Buyse et al., 2011) and predicted fewer externalizing problems among children exposed to harsh discipline at home (Hamre and Pianta, 2001; Mortensen and Barnett, 2018). In the most directly comparable work, Mortensen and Barnett (2019) found that teacher sensitivity was promotive for toddlers’ emotion regulation but not uniformly protective against intrusive parenting—a nuance suggesting that the compensation story is real but partial. These patterns align with a differential-susceptibility framing, in which certain children are disproportionately sensitive to environmental quality for better and for worse (Belsky and Pluess, 2009).

Extending this logic to the present investigation, we expect two distinguishable moderation patterns rather than one uniform effect. Where teachers provide high emotional support, children of emotion-coaching parents should gain ecological coherence: the affective scripts installed at home meet reinforcement at school, and regulatory growth accelerates. Where the same teachers meet children of emotion-dismissing parents, the predicted pattern is compensatory rather than amplifying, because high emotional support supplies alternative co-regulatory experiences that partially offset the shortfall in home-based coaching (Curby et al., 2009). Low emotional support, by contrast, should leave those children with fewer compensatory resources, deepening the negative association. This differentiated account, coherent as it is in theory, has not been tested directly—a gap the present study aims to close (Hajal and Paley, 2020).

Four hypotheses follow, stated at the level of constructs; how each construct was operationalized, and which covariates the models carry, is set out in the Method section. Hypothesis 1a holds that parental emotion coaching is prospectively and positively related to children’s adaptive classroom emotional regulation, and negatively related to their classroom lability/negativity. Hypothesis 1b holds the converse for parental emotion dismissing: a negative relation to adaptive regulation and a positive relation to lability/negativity. Hypothesis 2a holds that teacher emotional support moderates the coaching pathway in an amplifying direction, so that the coaching advantage is larger where classroom emotional support is high. Hypothesis 2b holds that teacher emotional support moderates the dismissing pathway in a compensatory direction, so that the disadvantage associated with dismissing is attenuated where classroom emotional support is high. Classroom organization and instructional support are examined as moderators as well, but without directional predictions, since their conceptual distance from the affective substrate of regulation is greater.

## 3 Research design and methods

### 3.1 Participants and procedures

The study followed a two-wave longitudinal panel design with a 12-month interval between assessments. Participants were 326 children (aged 6–8 years at baseline) and their primary caregivers recruited from six public elementary schools located across three adjacent districts of a large eastern-Chinese metropolitan area. All six schools operated under the same municipal-level curriculum guidelines and shared broadly comparable class sizes (35–45 children per class) and staffing patterns. Neighborhood socioeconomic profiles varied modestly: two schools drew from predominantly middle-income catchments, three from mixed catchments, and one from a neighborhood with a larger share of families receiving low-income subsidies (approximately 18% of enrolled students). Parental education, reported in Table 1, spanned high-school-or-below through postgraduate levels. All children were native Mandarin speakers, and no participating child was enrolled in a bilingual or minority-language track. Inclusion required enrolment in grades one or two, no diagnosed developmental or psychiatric disorder, and consent from at least one parent to participate in both waves.

**Table 1:** Demographic characteristics of participants.

Variable	Category	*n*	%
Child gender	Male	152	52.6
	Female	137	47.4
Child grade at T1	Grade 1	158	54.7
	Grade 2	131	45.3
Responding parent	Mother	231	79.9
	Father	58	20.1
Parental education	High school or below	74	25.6
	Bachelor’s degree	149	51.6
	Master’s degree or above	66	22.8

*N* = 289 child–parent dyads. Reporting parent refers to the caregiver who completed the MESQ at T1.

After accounting for attrition—mostly family relocation and incomplete questionnaire returns—the final analytic sample comprised 289 child–parent dyads, yielding a retention rate of 88.7%. A power analysis conducted before data collection confirmed that this sample size could detect medium-sized interaction effects in moderated regression models (Cohen, 1992). Because Chinese primary schools typically assign a single homeroom teacher (ban zhu ren) to a class for the duration of the first 2 years, every child in the analytic sample had known the rated teacher for at least 8 months at T2 (mean = 17.4 months, SD = 6.1). Table 1 summarizes the demographic profile of the sample. A recurring feature is that responding caregivers were predominantly mothers (79.9%). This distribution reflects the typical primary-caregiver structure in urban Chinese families and is common in the emotion-socialization literature (Chan et al., 2009; Han et al., 2015), but it does place a ceiling on how much the coaching and dismissing coefficients speak to paternal socialization. We return to this point in the Limitations section, and we report a sensitivity check restricted to mother-only dyads as a supplementary analysis.

**Figure 1:**
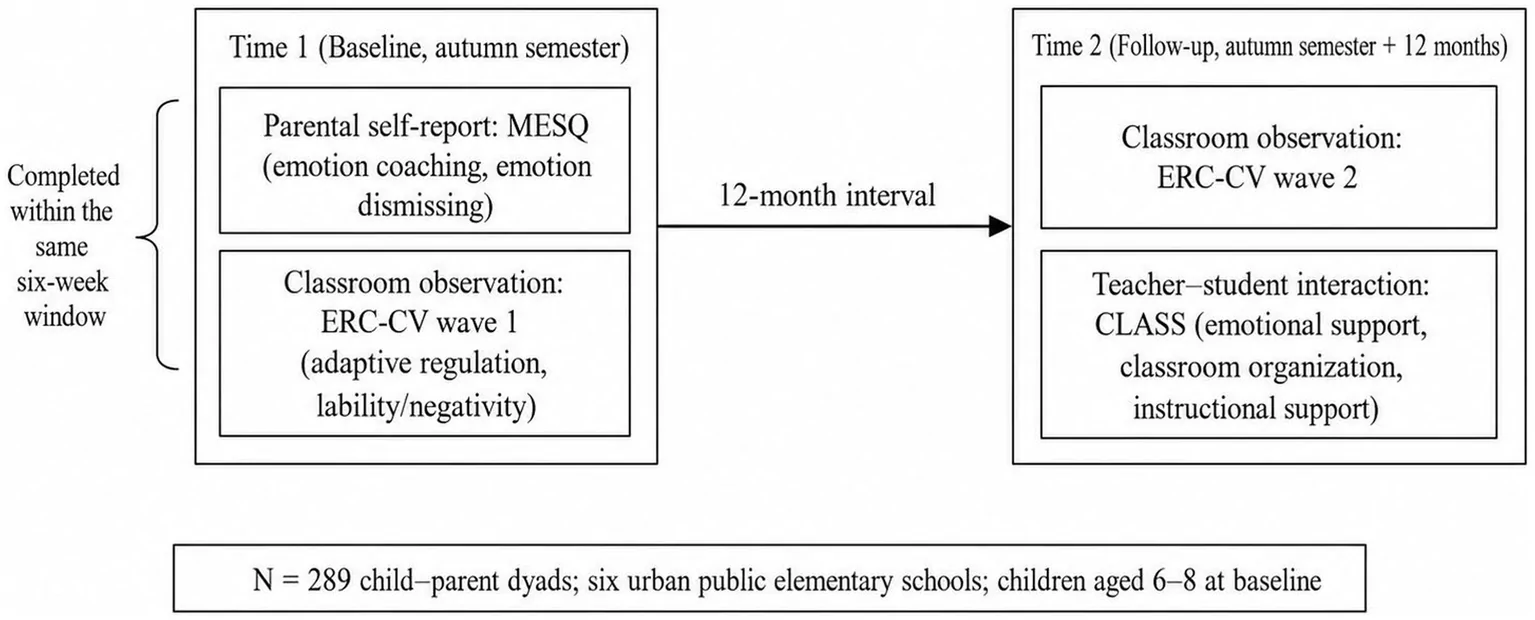
Longitudinal research design and data-collection sequence. The parental self-report and classroom observation modules within T1 were completed within the same six-week window.

At Time 1 (T1, autumn semester), parents completed self-report measures of meta-emotion philosophy and demographics, and trained observers rated children’s classroom emotional regulation during two standardized lesson observations per child. Twelve months later, at Time 2 (T2), the observational protocol for classroom emotional regulation was repeated with the same coding scheme, and homeroom teachers were concurrently rated on teacher–student interaction quality covering the intervening academic year. Figure 1 depicts the overall design; the two T1 data streams (parental self-report and classroom observation) occurred within the same six-week window and are grouped together as T1 assessments to make this timing explicit. All procedures received approval from the institutional ethics review board at the authors’ affiliated university; written informed consent was obtained from parents, and children provided age-appropriate verbal assent following SRCD guidelines for developmental research with minors (Society for Research in Child Development, 2021).

### 3.2 Research instruments and variable measurement

Parental meta-emotion philosophy was assessed with the Maternal Emotional Style Questionnaire (MESQ), a 14-item self-report instrument developed by Lagacé-Séguin and Coplan (2005) and subsequently adapted for Chinese populations (Qiu and Shum, 2022). The MESQ produces two subscale scores—emotion coaching and emotion dismissing—rated on a five-point Likert scale from 1 (*strongly disagree*) to 5 (*strongly agree*). A sample coaching item reads, “When my child is sad, I take time to try to understand what he/she is feeling,” and a sample dismissing item reads, “Children’s angry moods are usually best ignored so they pass quickly.” The Chinese adaptation used here retains the two-factor structure identified in the original validation, and prior applications with Chinese caregivers have reported acceptable internal consistency and sensitivity to change on both subscales (Qiu and Shum, 2022).

Two measurement decisions deserve an explicit rationale, since section 2.1 presented interview-based, questionnaire-based, and observational routes as genuine alternatives rather than as a settled hierarchy. First, parental beliefs were captured by questionnaire rather than by the Meta-Emotion Interview or by observational coding of parent–child emotion dialogues. The construct at stake is attitudinal rather than behavioral; a sample of this size could not have been interviewed and coded within the recruitment window; and the MESQ already has a validated Chinese adaptation, which an *ad hoc* coding scheme would have lacked. Second, and in order to keep predictor and outcome methodologically independent, children’s regulation was not solicited from the same informants but observed directly by trained coders. The cost of the first decision—susceptibility to social desirability—is real, and we return to it among the limitations; its compensation is that the two sides of the model do not share a reporter (Rogosa, 1980). Coaching and dismissing are treated as separate continuous predictors throughout, both to preserve information and because dual endorsement is well documented in Chinese samples (Fiorilli et al., 2015; Wang et al., 2019; Trevethan et al., 2021).

Children’s classroom emotional regulation was captured through structured behavioral observation using the Emotion Regulation Checklist–Classroom Version (ERC-CV), an observational adaptation of the Shields and Cicchetti ERC comprising 18 items across two empirically derived dimensions: lability/negativity (nine items; e.g., “recovers slowly after a disappointing outcome,” “shifts abruptly between excitement and sadness”) and adaptive regulation (nine items; e.g., “recovers composure quickly after a peer conflict,” “waits for a turn to speak even when eager”) (Shields and Cicchetti, 1997; Shields and Cicchetti, 1998). Every item is rated on a five-point scale, from 1 (*never characteristic of the child during the segment*) to 5 (*almost always characteristic*). Two rounds of standardized classroom observations were conducted per child at each wave, each round consisting of a 35-min recording of a mathematics or Chinese-language lesson. Cameras were positioned so as to capture the target child’s face and torso in the middle of the frame while remaining sensitive to peer and teacher interactions in the surround; a hand-held lavaliere microphone was worn by the target child in one of the two sessions.

The coding procedure ran as follows. Each 35-min lesson was divided into seven consecutive five-minute segments, so that every child contributed 14 segments per wave. Two trained raters, blind to the parental data and to each other’s ratings, rated all 18 items independently within every segment, and no rating was carried over from one segment to the next. A child’s score on an item at a given wave is the mean of that item across available segments and across the two raters; a subscale score is then the mean of its nine item scores, so that the metric remains the original five-point scale rather than a count of events.

Non-occurrence was governed by a rule agreed during training and applied consistently thereafter (Bakeman and Quera, 2011). When a behavior could have occurred but did not—no peer conflict arose, so no slow recovery was seen—the item was scored at the low-frequency end of the scale, since the absence of dysregulated behavior is itself informative. When a segment offered no occasion for the behavior at all, or when the child was momentarily off-camera, the item was marked “no opportunity” and excluded from that segment’s mean rather than scored as zero, which prevents structural absences from being read as competence. Children needed at least eight valid segments per wave to be retained, a criterion met by every child in the analytic sample (mean number of valid segments = 13.2, SD = 1.1). Coder training required a two-week workshop, calibration on 15 practice tapes, and a certification threshold of ICC ≥ 0.80. Inter-rater reliability, computed on 20% of the final sample as two-way random single-rater ICCs, ranged from 0.81 to 0.89 for the aggregated wave-level subscale scores and from 0.74 to 0.83 at the more demanding segment level.

**Figure 2:**
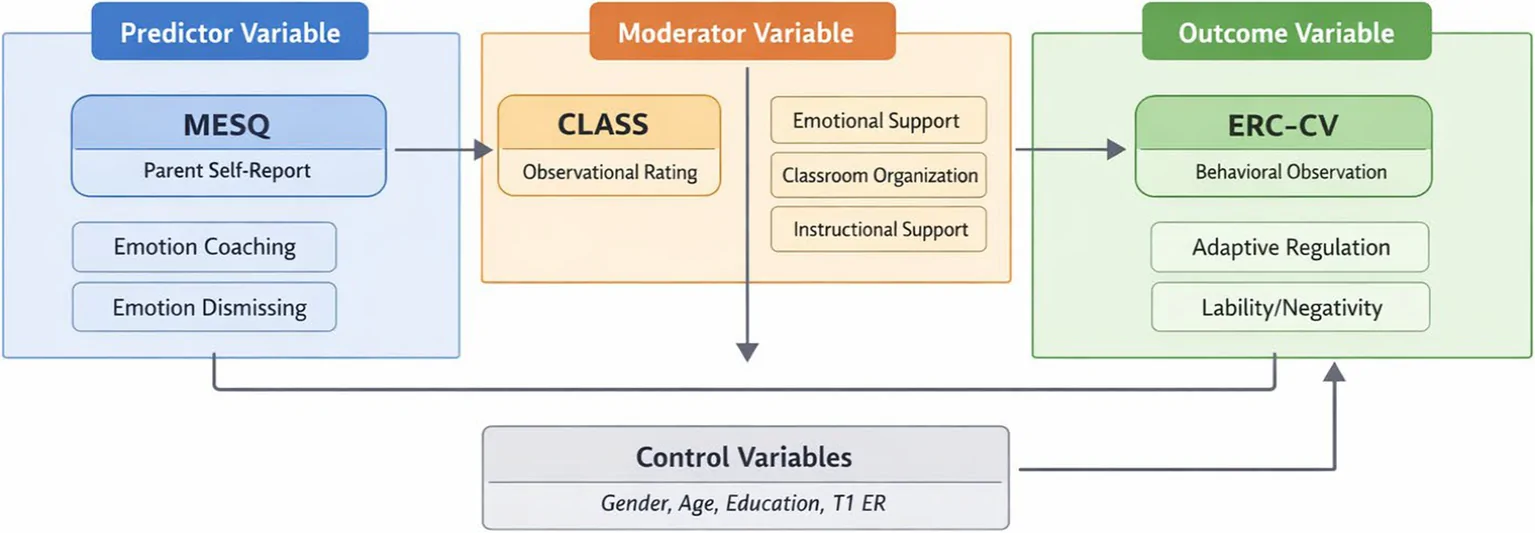
Conceptual framework of variable measurement. The framework links parental meta-emotion philosophy (predictor), teacher–student interaction quality (moderator), and children’s classroom emotional regulation (outcome), the latter comprising adaptive regulation and lability/negativity.

Teacher–student interaction quality was evaluated at T2 with the Classroom Assessment Scoring System (CLASS), a widely validated observational tool capturing three broad domains: emotional support (e.g., positive climate, teacher sensitivity), classroom organization (e.g., behavior management, productivity), and instructional support (e.g., concept development, quality of feedback) (Pianta et al., 2008). Certified CLASS observers rated each participating classroom on a seven-point scale following two 30-min observation cycles. Because CLASS was measured concurrently with the T2 outcome, its role in the design is moderational rather than temporally antecedent, and the direction of influence cannot be resolved on the basis of the present timing; we return to this point when interpreting effects. Figure 2 depicts the conceptual framework linking these measurement domains to the study’s predictor, outcome, and moderator variables.

Control variables included child gender, age at T1, parental education level, and baseline classroom emotional regulation. Gender and age were extracted from school administrative records; parental education was collected via the demographic questionnaire. Each outcome was adjusted for its own T1 score—adaptive regulation for T1 adaptive regulation, lability/negativity for T1 lability/negativity—so that the models capture change in regulatory functioning rather than static between-person differences (Rogosa, 1980). Table 2 summarizes internal consistency for each instrument. Every Cronbach’s α met or exceeded the conventional threshold of 0.70, and the CLASS instructional support subscale is reported alongside the other two domains.

**Table 2:** Internal consistency reliability coefficients for study instruments.

Instrument	Subscale/Domain	Cronbach’s α
MESQ	Emotion coaching	0.83
MESQ	Emotion dismissing	0.78
ERC-CV (T1)	Adaptive regulation	0.85
ERC-CV (T1)	Lability/negativity	0.82
ERC-CV (T2)	Adaptive regulation	0.87
ERC-CV (T2)	Lability/negativity	0.84
CLASS	Emotional support	0.91
CLASS	Classroom organization	0.88
CLASS	Instructional support	0.86

MESQ, Maternal Emotional Style Questionnaire; ERC-CV, Emotion Regulation Checklist–Classroom Version; CLASS, Classroom Assessment Scoring System.

### 3.3 Data analysis strategy

Before hypothesis testing, missing data were handled with full information maximum likelihood estimation, which produces unbiased parameter estimates under the missing-at-random assumption (Enders, 2010). Normality was evaluated through skewness and kurtosis; variables exceeding absolute values of 2.0 and 7.0, respectively, were subjected to robust standard-error correction. All continuous predictors were mean-centered before entry, which eases interpretation of the interaction terms (Aiken and West, 1991). Adaptive regulation and lability/negativity were modeled as two parallel outcomes, with the same predictors, covariates, and model sequence, so that the two facets can be read side by side rather than one standing in for the other.

Because children (level 1) were nested within homeroom classrooms (level 2)—with an average of 8.3 target children per class in the analytic sample—we estimated random-intercept multilevel models to accommodate non-independence. The design makes this more than a technical precaution: CLASS is rated once per classroom and therefore enters as a level-2 variable, so every meta-emotion × CLASS product is a cross-level interaction between an individual-level predictor and a classroom-level moderator. The unconditional model yielded an intraclass correlation of ICC = 0.17 for T2 adaptive regulation and ICC = 0.14 for T2 lability/negativity, confirming that a non-trivial share of variance sits between classrooms. In level-1/level-2 notation, the full model expresses the T2 outcome ( $Y_{T2}$ ) as a function of demographic covariates, the corresponding baseline score ( $Y_{T1}$ ), the mean-centered meta-emotion dimensions $X_{\text{coach}}$ and $X_{\text{dismiss}}$ , the mean-centered CLASS domains $W_{\mathrm{ES}}$ , $W_{\mathrm{CO}}$ , and $W_{\text{IS}}$ , and their multiplicative interactions. The relationship can be expressed as Equation 1.

$$Y_{T2,\mathrm{ij}}=\gamma_{00}+\gamma_{01}W_{j}+\sum \gamma_{k0}X_{k,\mathrm{ij}}+\sum \gamma_{k1}(X_{k,\mathrm{ij}}\times W_{j})+\mu_{0j}+\varepsilon_{\mathrm{ij}}$$ (1)

The same specification was fitted separately to each of the two outcomes, so the equation is stated once and not repeated below. Estimation proceeded in three steps. Step 1 fitted an intercept-and-controls model with the T1 score of the outcome in question, demographics, and random class intercepts. Step 2 added the mean-centered main effects of the two meta-emotion dimensions and the three CLASS domains. Step 3 added the six cross-level interaction terms (two meta-emotion dimensions × three CLASS domains) that carry the moderation hypotheses. Significant interactions were probed with simple slopes at ±1 SD of the moderator, following Aiken and West (1991) and the multilevel extension described by Bauer and Curran (2005) and Preacher et al. (2006).

**Figure 3:**
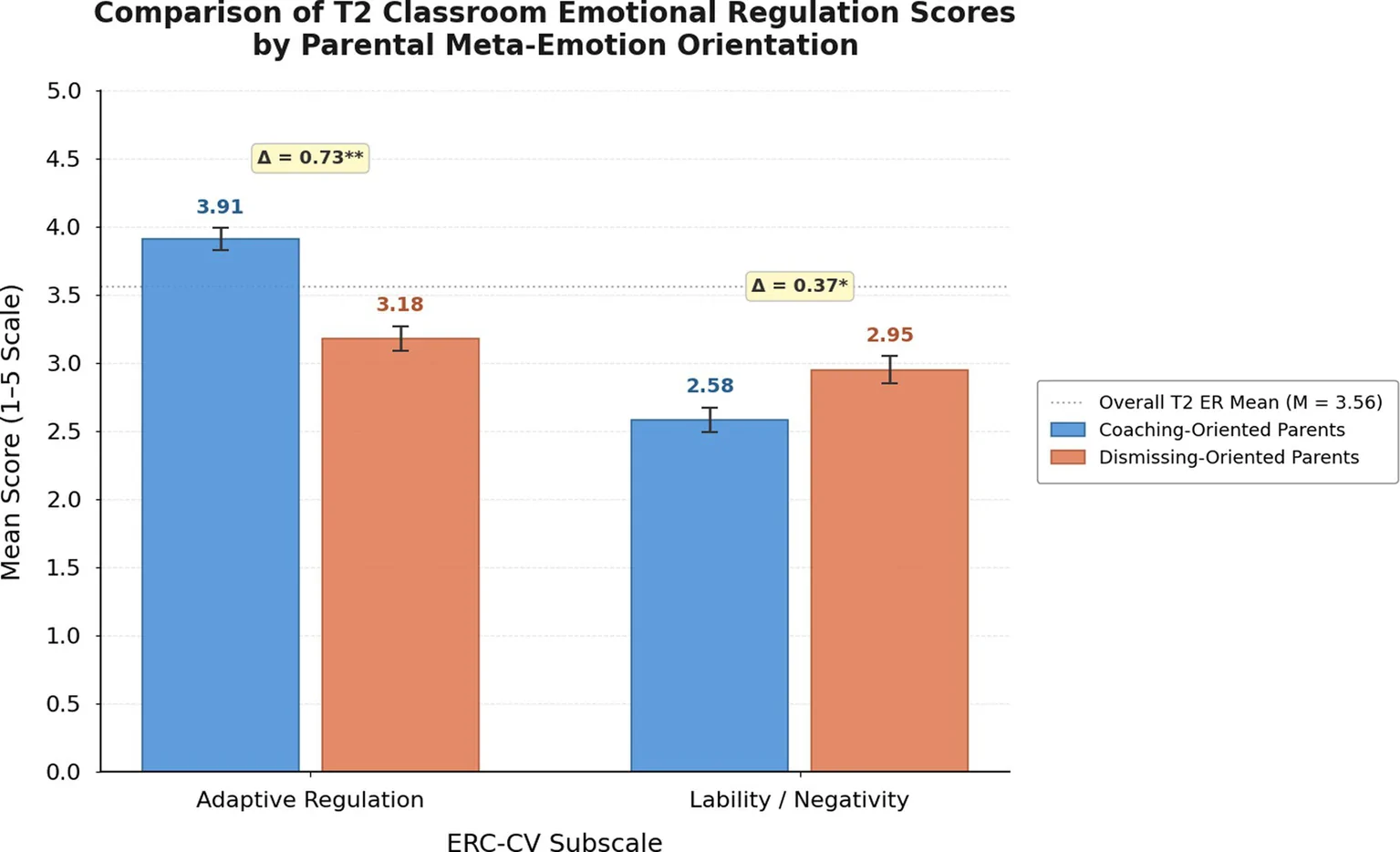
T2 classroom emotional regulation by parental predominant meta-emotion orientation. Groups were formed by a median split on the coaching–dismissing difference score. Because the split reduces two continuous dimensions to two categories, the figure is retained for reader orientation only; all inferential models use the continuous dimensions.

**Figure 4:**
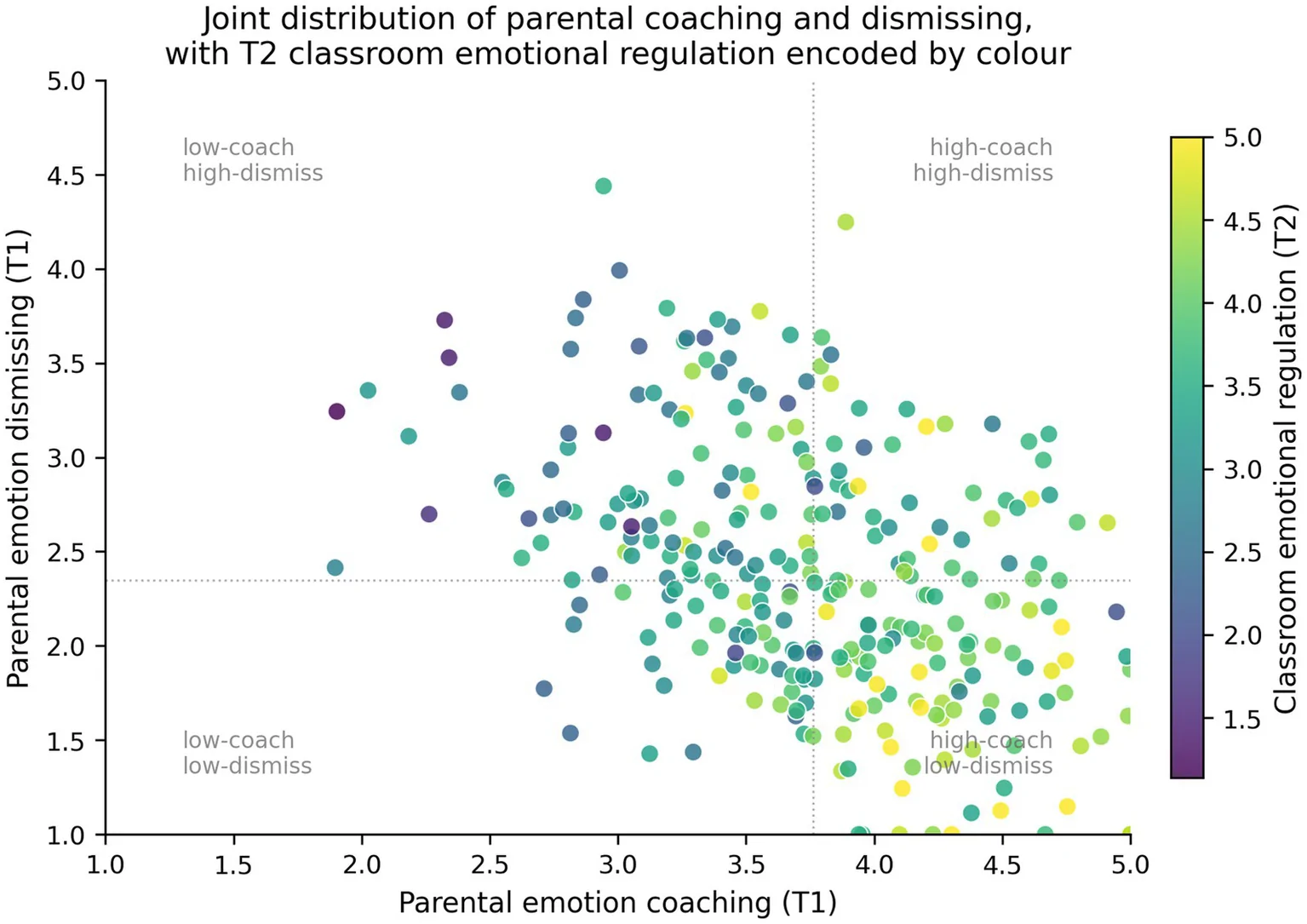
Joint distribution of parental emotion coaching and emotion dismissing at T1. T2 classroom emotional regulation is encoded as a color gradient. Dotted lines mark the sample median on each dimension. The scatter makes the dual-endorsement pattern visible and shows that outcomes vary continuously across the joint space rather than following a strict categorical split.

To keep the presentation compact, and to allow comparison with the earlier literature, the corresponding single-level OLS estimates are reported alongside the multilevel results in the tables that follow; substantive conclusions were identical in the two frameworks. For visual purposes only, children were additionally grouped by their parents’ predominant orientation via a median split on the difference score between the coaching and dismissing subscales. Because 42 participants (14.5%) sat within ±0.25 SD of the median cut, that grouping is intrinsically noisy; it produces the descriptive contrast in Figure 3 and nothing else. Figure 4 supplements the comparison with a two-dimensional scatter that keeps both dimensions on their original continuous scales, which is more faithful to the analytic strategy. All analyses were run in R 4.3.1 using *lme4* and *interactions*, with significance set at *p* < 0.05 (two-tailed).

## 4 Results

### 4.1 Descriptive statistics and correlation analysis

Table 3 presents means, standard deviations, response-scale ranges, and bivariate Pearson correlations among all major study variables, including both facets of classroom emotional regulation at both waves. Parents reported moderately high emotion-coaching scores and relatively low emotion-dismissing scores, which points to a generally coaching-oriented sample. Children’s adaptive regulation rose from T1 to T2 while their lability/negativity fell, both consistent with expected developmental gains across a 12-month interval. Among the CLASS domains, emotional support received the highest mean rating, followed by classroom organization and instructional support.

**Table 3:** Descriptive statistics and Pearson correlations for major study variables.

Variable	*M*	SD	Range	1	2	3	4	5	6	7	8
1. Emotion Coaching (T1)	3.72	0.64	1–5	—							
2. Emotion dismissing (T1)	2.41	0.71	1–5	−0.39**	—						
3. Classroom ER T1 (adaptive)	3.18	0.82	1–5	0.34**	−0.28**	—					
4. Classroom ER T1 (lability)	2.86	0.75	1–5	−0.31**	0.30**	−0.55**	—				
5. Classroom ER T2 (adaptive)	3.56	0.79	1–5	0.41**	−0.33**	0.58**	−0.42**	—			
6. Classroom ER T2 (lability)	2.61	0.72	1–5	−0.29**	0.27**	−0.40**	0.52**	−0.61**	—		
7. Emotional support (T2)	4.85	0.93	1–7	0.12*	−0.09	0.22**	−0.17**	0.36**	−0.28**	—	
8. Classroom organization (T2)	4.52	0.88	1–7	0.08	−0.06	0.19**	−0.14*	0.29**	−0.21**	0.61**	—
9. Instructional support (T2)	4.12	0.91	1–7	0.05	−0.04	0.14*	−0.09	0.18**	−0.12*	0.54**	0.47**

*N* = 289. ER, Emotional Regulation; Adaptive, adaptive regulation subscale; Lability, lability/negativity subscale. Response-scale ranges are those of the original instruments.*p < 0.05; **p < 0.01.

The correlation pattern already anticipates the multivariate story. Emotion coaching and emotion dismissing were moderately negatively related but far from redundant, consistent with the dual-endorsement literature in Chinese samples (Fiorilli et al., 2015; Wang et al., 2019). The two regulation facets correlated negatively with one another, yet the association fell well short of the near-unity that would make separate modeling pointless—a first indication that the lability side carries information the adaptive side does not. Emotional support correlated positively with T2 adaptive regulation and negatively with T2 lability/negativity, whereas classroom organization and, especially, instructional support showed weaker relations with both facets.

For a descriptive comparison, we grouped children by the predominant meta-emotion orientation of their parents. Figure 3 shows that children of coaching-oriented parents scored higher than their peers on T2 adaptive regulation, with the gap widening on the adaptive subscale but attenuated on lability/negativity. Because a median split of two continuous dimensions loses information and forces a false dichotomy on parents who scored close to the cut, Figure 4 supplements this contrast with a scatter that keeps both dimensions on their original scale and encodes T2 regulation as a color gradient. The scatter makes visible a dispersion the bar chart hides: some parents endorsed both coaching and dismissing at above-average levels, and their children’s T2 scores are neither uniformly high nor uniformly low, which indicates that the joint distribution—not either extreme alone—carries the developmental signal.

### 4.2 Predictive analysis of parental meta-emotion philosophy on children’s classroom emotional regulation

Hierarchical regression tested whether parental meta-emotion philosophy at T1 was prospectively associated with children’s classroom emotional regulation at T2, after accounting for demographic covariates and the baseline level of the outcome. Table 4 reports the models for adaptive regulation and Table 5 the parallel models for lability/negativity. In Step 1, control variables explained 35% of the variance in T2 adaptive regulation and 31% of the variance in T2 lability/negativity, the corresponding T1 score being by some distance the strongest predictor in each case. Adding the meta-emotion dimensions in Step 2 produced significant increments of 5 and 4%, respectively.

**Table 4:** Hierarchical regression predicting T2 adaptive classroom emotional regulation.

Predictor	Step 1 *B*	Step 1 β	Step 1 *p*	Step 2 *B*	Step 2 β	Step 2 *p*
Gender	0.04	0.03	0.621	0.03	0.02	0.714
Age	0.09	0.07	0.198	0.07	0.06	0.273
Parental education	0.11	0.10	0.068	0.08	0.07	0.175
Classroom ER adaptive (T1)	0.52	0.54	< 0.001	0.44	0.46	< 0.001
Emotion coaching	—	—	—	0.21	0.17	0.002
Emotion dismissing	—	—	—	−0.15	−0.13	0.018
$R^{2}$ / $\Delta R^{2}$		0.35			0.40/0.05**	

β , standardized regression coefficient.**p < 0.01. Parallel multilevel estimation with random class intercepts yielded coefficients within 0.02 of the OLS values shown here.

**Table 5:** Hierarchical regression predicting T2 classroom lability/negativity.

Predictor	Step 1 *B*	Step 1 β	Step 1 *p*	Step 2 *B*	Step 2 β	Step 2 *p*
Gender	−0.06	−0.05	0.402	−0.05	−0.04	0.469
Age	−0.05	−0.04	0.451	−0.04	−0.03	0.552
Parental education	−0.09	−0.08	0.132	−0.07	−0.06	0.241
Classroom ER lability (T1)	0.49	0.51	< 0.001	0.43	0.45	< 0.001
Emotion coaching	—	—	—	−0.13	−0.11	0.041
Emotion dismissing	—	—	—	0.17	0.15	0.009
$R^{2}$ / $\Delta R^{2}$		0.31			0.35/0.04**	

β , standardized regression coefficient. Higher scores indicate greater lability/negativity.**p < 0.01. Parallel multilevel estimation with random class intercepts yielded coefficients within 0.02 of the OLS values shown here.

The coefficients ran in the predicted directions on both facets. Emotion coaching was positively associated with T2 adaptive regulation and negatively, though more weakly, with T2 lability/negativity; emotion dismissing showed the mirror-image pattern. Hypotheses 1a and 1b are therefore supported on both facets, with the qualification that coaching bears more strongly on the constructive side of regulation than on the dysregulated side—an asymmetry we take up in the Discussion. Substantively, what parents believe about emotion continues to relate to change in children’s classroom regulatory behavior across a 12-month interval even once prior regulatory levels are held constant.

**Figure 5:**
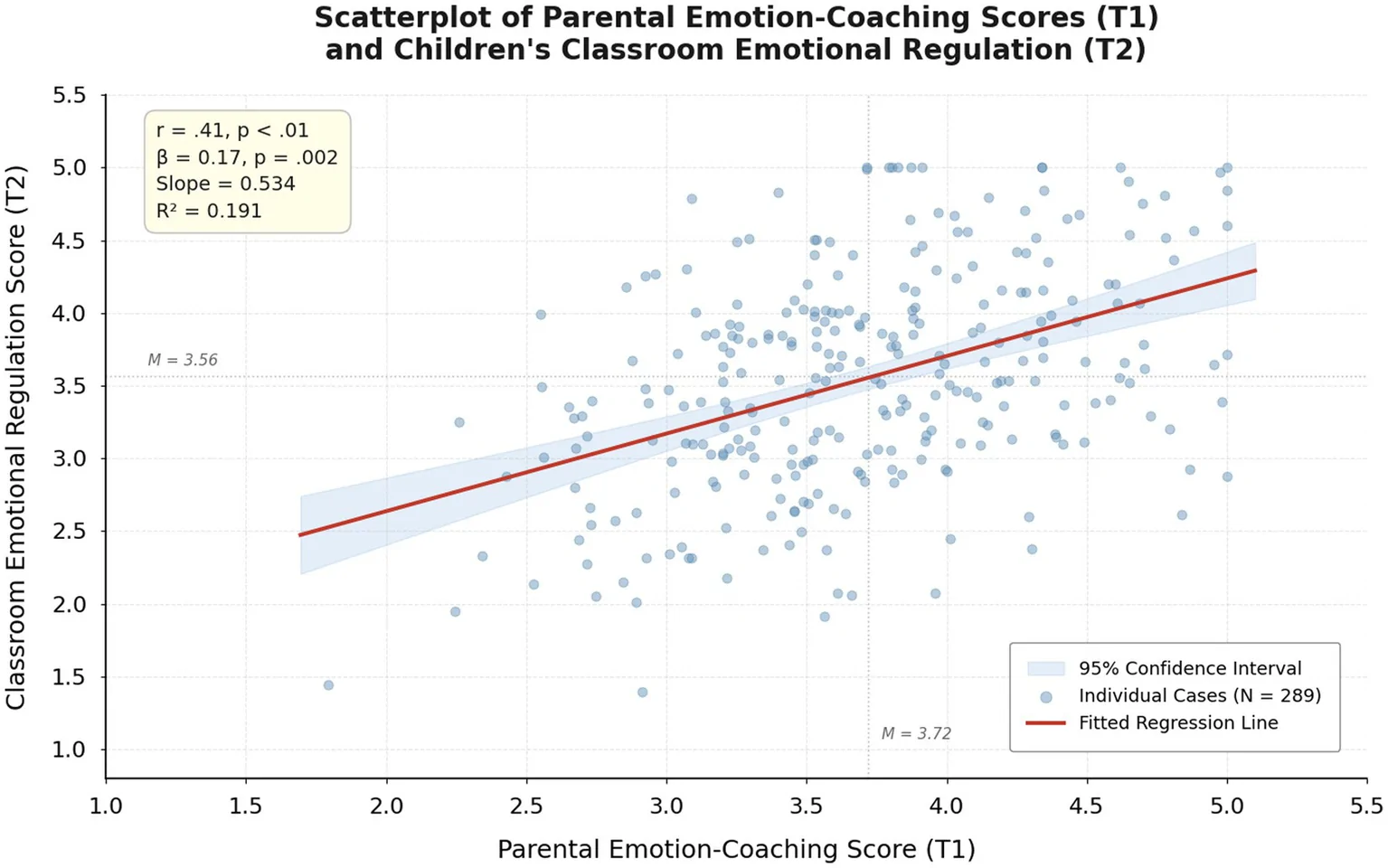
Parental emotion coaching at T1 and children’s adaptive classroom regulation at T2. The line is the fitted regression slope. Substantial dispersion around it motivates the moderation analyses reported in section 4.3.

To quantify the unique predictive contribution of the meta-emotion block, we computed Cohen’s $f^{2}$ from the standard formula for the incremental effect size, $f^{2}$ = ( $R^{2}$ full − $R^{2}$ reduced)/(1 − $R^{2}$ full). This yielded $f^{2}$ = 0.083 for adaptive regulation and $f^{2}$ = 0.062 for lability/negativity, both small-to-medium. Given that the T1 score had already absorbed a substantial share of the outcome variance, these increments are worth taking seriously rather than dismissing as trivial. Figure 5 depicts the association between parental emotion-coaching scores and T2 adaptive regulation via a scatter with a fitted regression line. A clear positive linear trend is visible, but considerable individual dispersion remains around the fitted slope—dispersion that section 4.3 shows to be partly attributable to classroom-level moderation.

### 4.3 Testing the moderating effect of teacher–student interaction

To examine whether teacher–student interaction quality moderated the association between parental meta-emotion philosophy and T2 classroom emotional regulation, the interaction terms were entered as a third step of the multilevel model described in section 3.3. Both meta-emotion dimensions were crossed with all three CLASS domains, producing six interaction terms per outcome. Table 6 reports the full moderation model for adaptive regulation and for lability/negativity side by side.

**Table 6:** Multilevel regression testing the moderating effect of teacher–student interaction quality.

Predictor	Adaptive regulation				Lability/negativity			
	*B*	SE	β	*p*	*B*	SE	β	*p*
Baseline regulation (T1)	0.39	0.06	0.41	< 0.001	0.38	0.06	0.40	< 0.001
Emotion coaching	0.18	0.07	0.15	0.006	−0.12	0.06	−0.10	0.048
Emotion dismissing	−0.14	0.06	−0.12	0.022	0.16	0.06	0.14	0.011
Emotional support	0.17	0.04	0.20	< 0.001	−0.13	0.04	−0.16	0.002
Classroom organization	0.07	0.05	0.08	0.142	−0.05	0.05	−0.06	0.284
Instructional support	0.03	0.04	0.04	0.431	−0.02	0.04	−0.02	0.663
Coaching × Emotional support	0.12	0.05	0.11	0.026	−0.06	0.05	−0.05	0.283
Dismissing × Emotional support	0.10	0.05	0.10	0.038	−0.11	0.05	−0.10	0.043
Coaching × Classroom organization	0.05	0.06	0.04	0.413	−0.03	0.05	−0.03	0.551
Dismissing × Classroom organization	0.03	0.05	0.03	0.524	−0.04	0.05	−0.04	0.462
Coaching × Instructional support	0.04	0.05	0.04	0.462	−0.02	0.05	−0.02	0.701
Dismissing × Instructional support	0.02	0.05	0.02	0.719	−0.01	0.05	−0.01	0.812
$R^{2}$ (marginal/conditional)	0.43/0.52				0.36/0.45			

*N* = 289 children nested in 35 classrooms; the two outcomes were modeled separately. Random intercepts for classroom; ICC = 0.17 for adaptive regulation and 0.14 for lability/negativity. Baseline (T1) refers to the T1 score of the corresponding outcome. The CLASS domains are classroom-level (level-2) variables, so every product term is a cross-level interaction. Controls (gender, age, parental education) suppressed for space. β , standardized regression coefficient. Multilevel and single-level OLS yielded qualitatively identical inference for every term shown.

Three interactions reached significance, and their distribution is instructive. Consistent with Hypothesis 2a, emotion coaching × emotional support was positive for adaptive regulation: the predictive effect of coaching was stronger in classrooms rated higher on emotional support. Consistent with Hypothesis 2b, emotion dismissing × emotional support dampened the unfavorable slope of dismissing on both facets—positive for adaptive regulation, negative for lability/negativity—which is precisely what a buffering pattern looks like when two outcomes are scored in opposite directions. Coaching, by contrast, was not significantly moderated on the lability facet. The remaining interactions, all of them involving classroom organization or instructional support, were small and non-significant. This selective pattern supports the argument advanced in section 2.3 that emotional support carries the affective-scaffolding function most directly analogous to what coaching parents provide.

**Figure 6:**
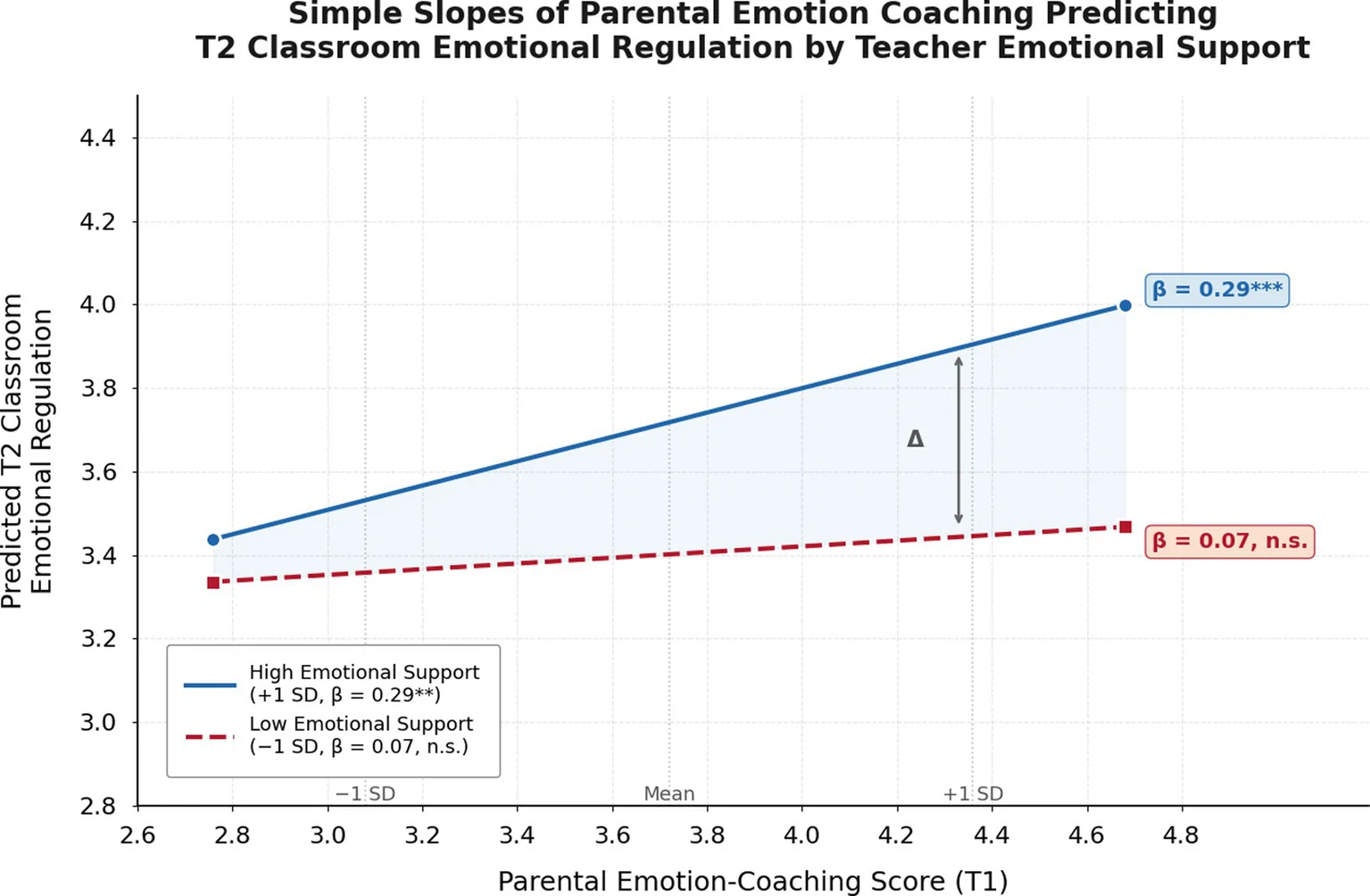
Simple slopes of parental emotion coaching predicting T2 adaptive regulation at high and low teacher emotional support. Emotional support is evaluated at ±1 SD. The positive coaching–regulation link is robust at high emotional support and attenuates to non-significance at low emotional support.

**Figure 7:**
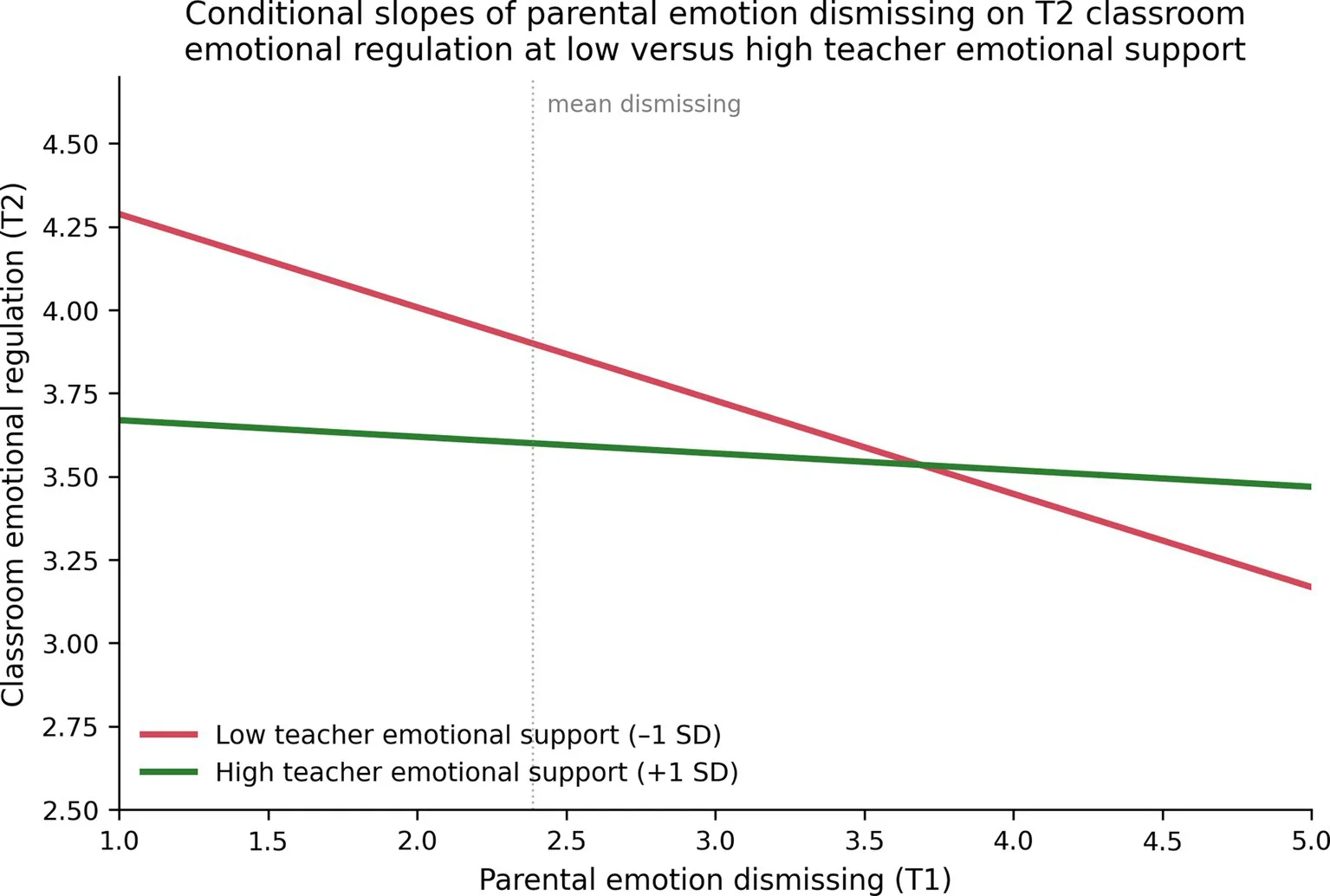
Simple slopes of parental emotion dismissing predicting T2 adaptive regulation at high and low teacher emotional support. Emotional support is evaluated at ±1 SD. The negative dismissing–regulation slope is meaningful only when teacher emotional support is low, which is consistent with a compensatory buffering pattern.

To decompose the significant interactions, simple slopes were estimated at ±1 *SD* of teacher emotional support. For emotion coaching predicting adaptive regulation, the conditional slope was robust at high emotional support ( β = 0.29, *t* = 3.84, *p* < 0.001) but fell to non-significance at low emotional support ( β = 0.07, *t* = 1.02, *p* = 0.309); the coaching advantage is thus visible essentially only where emotional support lies above the sample mean, as Figure 6 shows. For emotion dismissing predicting adaptive regulation, the conditional slope was negative and significant at low emotional support ( β = − 0.24, *t* = −2.71, *p* = 0.007) but small and non-significant at high emotional support ( β = − 0.05, *t* = −0.63, *p* = 0.530); Figure 7 displays that contrast.

**Figure 8:**
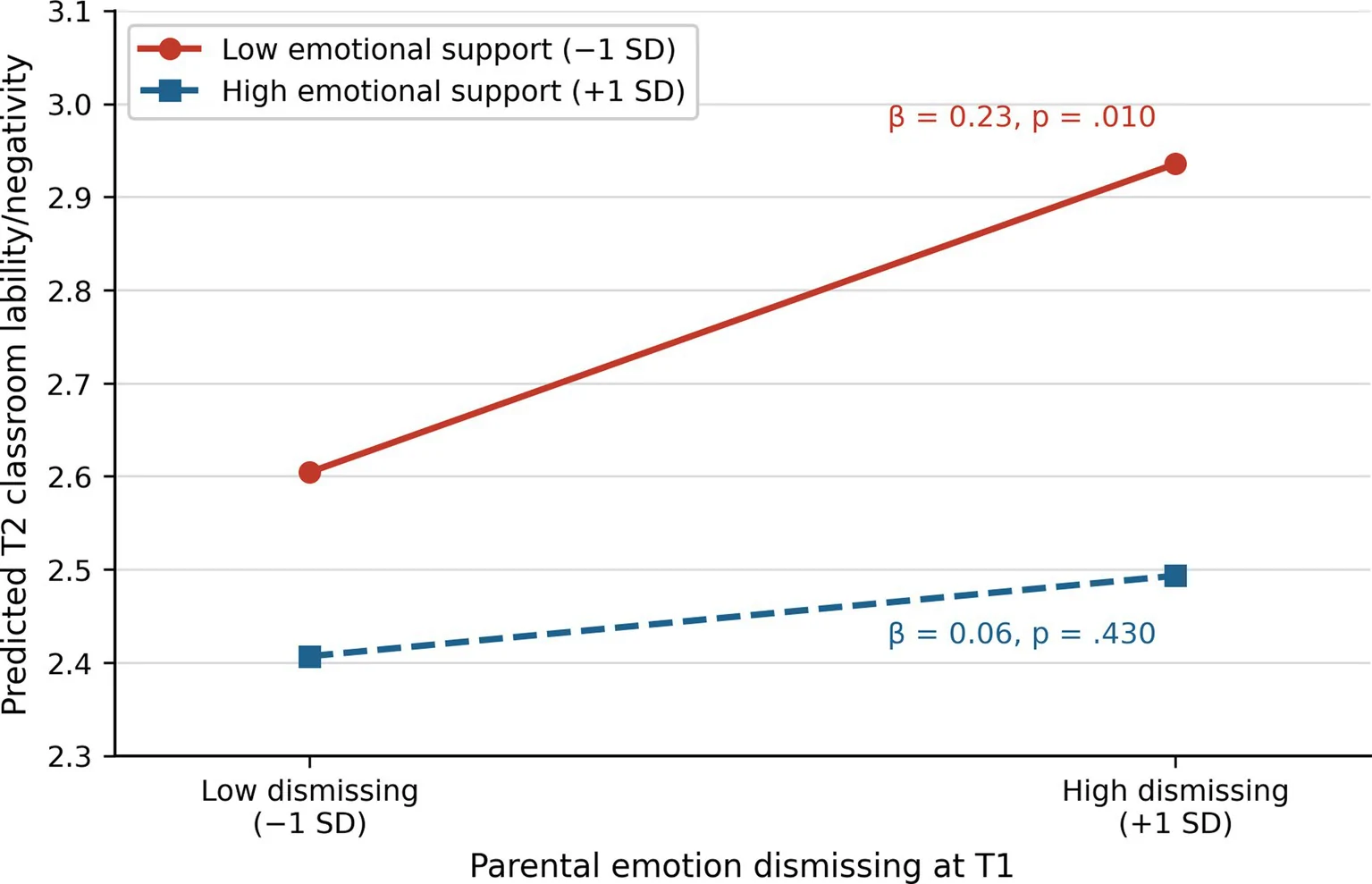
Simple slopes of parental emotion dismissing predicting T2 lability/negativity at high and low teacher emotional support. Emotional support is evaluated at ±1 SD; higher values on the vertical axis indicate greater lability/negativity. The positive dismissing–lability slope is meaningful only in classrooms low in emotional support, mirroring the buffering pattern reported for adaptive regulation in Figure 7.

The lability facet tells the same story from the other side. Dismissing predicted greater lability/negativity where emotional support was low ( β = 0.23, *t* = 2.58, *p* = 0.010), whereas the slope was negligible where emotional support was high ( β = 0.06, *t* = 0.79, *p* = 0.430); Figure 8 plots the contrast. Taken together, the coaching-amplification and dismissing-buffering patterns show that one and the same source of school-side quality can play two quite different developmental roles, depending on which family-level orientation it happens to meet.

## 5 Discussion

The study asked whether parental meta-emotion philosophy is prospectively associated with children’s classroom emotional regulation, and whether teacher–student interaction quality conditions that association. Read together, the four hypotheses received support, though not with uniform strength. Coaching and dismissing at T1 forecast change in both facets of T2 regulation in opposite directions, and teacher emotional support moderated the family-level pathways—amplifying the coaching link on the adaptive facet and buffering the dismissing link on both. Because CLASS was measured concurrently with the T2 outcome, we frame these findings as associations conditional on classroom quality rather than as causal effects of it. The direction of influence between teacher responsiveness and observed regulation cannot be resolved on the present timing, and it remains quite plausible that classrooms full of well-regulated children elicit warmer teacher behavior as much as the reverse.

The positive coaching–regulation association fits comfortably with an internalization account developed most fully by Morris et al. (2007), Morris et al. (2017), and Denham et al. (2015). When parents treat a child’s negative affect as an occasion for guided reflection—naming the feeling, validating its legitimacy, and collaboratively exploring coping options—they scaffold an internal working model of affect management that children carry beyond the home. The classroom then supplies a set of situations in which this model must actually run: managing frustration when a maths problem yields no traction, waiting for a turn in group discussion, absorbing corrective feedback without defensive escalation. What transfers is less a discrete skill than an orientation—a tendency to treat negative affect as informative rather than as threat—and this orientation held up across a context structurally quite different from where it was acquired (Blair and Raver, 2015).

An asymmetry within our own results sharpens this reading. Coaching predicted the adaptive facet more strongly than it predicted lability/negativity, and the conceptual refinement introduced in section 2.1 offers a plausible account of why. The coaching construct blends an affect-acceptance component with an affect-shaping component that teaches appropriate expression and, at times, restraint. Both components should build the constructive repertoire that adaptive regulation captures; on the dysregulated facet, however, they pull against each other, since acceptance licenses expression while shaping curbs it. A combined subscale therefore dilutes what may be two opposing signals, and an instrument that separated the components would be a natural next step for this literature.

The negative association with emotion dismissing, though smaller in magnitude, was substantively distinct. Parents who consistently minimize or redirect affective expression communicate that emotion is unmanageable or socially inappropriate, and this message tracks into children’s classroom behavior in the form of suppression as a default strategy—a strategy that laboratory work has repeatedly linked to physiological cost and cognitive-resource depletion (Eisenberg et al., 1998). The child who learns at home to hide sadness rather than talk about it may hesitate to ask for help when confused, or may escalate minor frustration into behavioral disruption because no constructive pathway has been modeled. The lability results make the same case from the other direction: dismissing predicted precisely the profile of abrupt shifts and slow recovery that suppression as a default would produce, and that profile is the one most consistently tied to lost classroom productivity (Graziano et al., 2007). Our finding that the association is attenuated in emotionally supportive classrooms is not merely descriptive; it says something about mechanism. Emotional support supplies what dismissing parents do not: an adult who names and receives affect. That secondary attachment work has been argued for in the theoretical literature (Verschueren and Koomen, 2012; Sabol and Pianta, 2012) and, more directly, in empirical demonstrations of teacher sensitivity buffering early family risk (Buyse et al., 2011; Mortensen and Barnett, 2018; Mortensen and Barnett, 2019).

The moderation results deserve to be examined as a pair, because the coaching-amplification and dismissing-buffering patterns come from the same interaction structure and therefore describe two faces of a single mechanism. Where the affective messages at home and at school are congruent—both environments valuing emotional openness and guided regulation—the developmental signal is reinforced. Where the home environment offers less coaching but the school compensates, some of the loss is absorbed. Where neither side offers affective scaffolding, the cost accumulates on both facets at once: dismissing predicts weak adaptive regulation and high lability most strongly in low-support classrooms. This pattern echoes Mortensen and Barnett’s toddler findings that teacher sensitivity is promotive but not uniformly protective (Mortensen and Barnett, 2019), and it fits the differential-susceptibility framing more generally (Belsky and Pluess, 2009), in which environmental quality amplifies whatever the child has already been given.

The non-significant interactions with classroom organization and instructional support are informative in their own right. Well-organized classrooms clearly support behavioral compliance and routine adherence, and instructional support enhances cognitive engagement; but the regulatory demands captured by the ERC-CV outcome—managing frustration, modulating excitement, recovering from disappointment—are more fundamentally relational than procedural or cognitive (Pianta et al., 2008; Mashburn et al., 2008). It fits, then, that emotional support, which directly addresses the affective dimension of the teacher–child exchange, would interact with a family-level affective variable in ways that organizational structure or cognitive scaffolding do not. Reporting all three moderators, rather than only the significant one, is essential for this reading: it is the *difference* across CLASS domains, not any single domain in isolation, that supports the mechanistic argument.

Placed within a cross-cultural frame, the findings speak to two tensions in the emotion socialization literature that Raval and colleagues have recently articulated (Fiorilli et al., 2015; Wang et al., 2019; Raval, 2023; Trevethan et al., 2021). First, the coaching–dismissing typology was developed with Euro-American families for whom the two orientations tend to be relatively polarized, and Fiorilli et al. (2015) have documented that Chinese mothers score higher than Italian mothers on both subscales of the MRCES. Modeling coaching and dismissing as separate continuous dimensions, as we did, preserves the empirical possibility that a given Chinese parent endorses both; median-split typologies obscure this. Second, the modest overall effect sizes may partly reflect the fact that Chinese norms around emotional restraint dilute the coaching–dismissing contrast relative to Western samples. What our data show, nonetheless, is that even within a context where dismissing is not universally maladaptive, the coaching orientation confers measurable classroom-regulatory advantages; the core mechanism—parental engagement with children’s affect as a teachable domain—appears to operate across cultural boundaries even if its magnitude varies.

A final observation cuts across the two moderation findings. Family and school do not exert independent, additive effects on regulatory development; they appear to function as an interconnected system whose joint configuration matters more than either component alone. For children whose parents are already coaching-oriented, a classroom rich in emotional support creates a developmental amplifier; for children whose home environment offers less emotional guidance, a supportive teacher partially compensates but does not fully restore the trajectory. This interdependence highlights the need for coordinated intervention approaches that address family and school in concert rather than each in isolation—a point developed further in the concluding section.

## 6 Conclusion

Across a two-wave panel of 289 child–parent dyads followed over 12 months, four findings emerged with reasonable clarity. First, parental emotion coaching at baseline was positively associated with change in children’s adaptive classroom regulation 1 year later, and negatively—though more weakly—with change in lability/negativity, after adjusting for prior regulation, demographics, and classroom clustering. Second, parental emotion dismissing showed the mirror-image pattern on both facets. Third, teacher emotional support amplified the coaching link on the adaptive facet and buffered the dismissing link on both, whereas classroom organization and instructional support moderated neither. Fourth, and cutting across the previous three, coaching and dismissing behaved as separable continuous dimensions in this Chinese sample, consistent with cross-cultural evidence that Global-North taxonomies collapse variation these families do not (Raval, 2023).

Theoretically, the findings extend meta-emotion accounts beyond global socioemotional outcomes to context-specific regulatory behavior inside the classroom, a domain previously underexamined in this literature. They also give a concrete operationalization to bioecological claims about mesosystem interdependence between family and school microsystems (Bronfenbrenner and Morris, 2006). From a practical standpoint, parent education programs might benefit from teaching affect labeling, validation, and collaborative problem-solving with explicit reference to how these practices equip children for classroom demands. For schools, the results underscore that teacher emotional support is not decorative: it operates as a functional moderator of family-origin developmental influences, both amplifying advantage and offsetting risk. Professional development that strengthens teachers’ capacity to respond sensitively to children’s affect may therefore carry benefits that reach beyond the classroom door.

Several limitations temper these conclusions. Most consequentially, teacher–student interaction quality was assessed concurrently with the T2 outcome, so the moderation results describe an association conditional on classroom quality rather than a causal effect of classroom quality on regulation; reverse and reciprocal pathways cannot be excluded on this timing. Second, parental meta-emotion philosophy was captured through self-report, which invites social-desirability bias that observational or interview-based measurement would reduce (Baker et al., 2011). Third, both regulation facets were coded by the same trained observers, so they share a method even though they are independent of the parental report. Fourth, responding caregivers were predominantly mothers (79.9%); the coaching and dismissing coefficients therefore speak primarily to maternal socialization, and the paternal contribution—which prior work suggests has a distinct emotional profile in Chinese families (Wang et al., 2019)—is only indirectly represented here. Fifth, the sample was drawn from a single urban metropolitan area, which constrains generalizability to rural or socioeconomically more diverse populations. Finally, two time points cannot rule out unmeasured third-variable confounds, though the longitudinal design and baseline adjustment reduce the most obvious ones.

Three directions for future work stand out. Multi-wave designs spanning three or more time points would permit growth-curve modeling and a finer-grained analysis of developmental trajectories, and would allow the moderator itself to be measured before the outcome so that causal claims become defensible. Incorporating multiple informants—teachers, peers, and children themselves—alongside behavioral observation would supply a less method-dependent picture of classroom regulation, and separating the acceptance and shaping components of coaching would test the asymmetry reported here. Most importantly, randomized intervention trials that train parents in emotion coaching while simultaneously enhancing teacher emotional support could establish whether the amplification and buffering patterns documented here are amenable to deliberate cultivation, moving the field from prediction to actionable developmental change.

## Data Availability

The de-identified data that support the findings of this study, along with the analytic code, are available from the corresponding author on reasonable request.

## References

[ref1] Adrian M. Zeman J. Veits G. (2011). Methodological implications of the affect revolution: a 35-year review of emotion regulation assessment in children. J. Exp. Child Psychol. 110, 171–197. doi: 10.1016/j.jecp.2011.03.009, 21514596

[ref2] Aiken L. S. West S. G. (1991). Multiple Regression: Testing and Interpreting Interactions. Newbury Park, CA, United States: Sage.

[ref3] Bakeman R. Quera V. (2011). Sequential Analysis and Observational Methods for the Behavioral Sciences. Cambridge, United Kingdom: Cambridge University Press.

[ref4] Baker J. K. Fenning R. M. Crnic K. A. (2011). Emotion socialization by mothers and fathers: coherence among behaviors and associations with parent attitudes and children's social competence. Soc. Dev. 20, 412–430. doi: 10.1111/j.1467-9507.2010.00585.x, 21532915 PMC3082208

[ref5] Bauer D. J. Curran P. J. (2005). Probing interactions in fixed and multilevel regression: inferential and graphical techniques. Multivar. Behav. Res. 40, 373–400. doi: 10.1207/s15327906mbr4003_5, 26794689

[ref6] Belsky J. Pluess M. (2009). Beyond diathesis stress: differential susceptibility to environmental influences. Psychol. Bull. 135, 885–908. doi: 10.1037/a0017376, 19883141

[ref7] Blair C. Raver C. C. (2015). School readiness and self-regulation: a developmental psychobiological approach. Annu. Rev. Psychol. 66, 711–731. doi: 10.1146/annurev-psych-010814-015221, 25148852 PMC4682347

[ref8] Bronfenbrenner U. Morris P. A. (2006). “The bioecological model of human development,” in Handbook of child Psychology: Vol. 1. Theoretical Models of human Development, eds. Lerner R. M. Damon W.. 6th ed (Hoboken, NJ, United States: Wiley), 793–828.

[ref9] Buyse E. Verschueren K. Doumen S. (2011). Preschoolers' attachment to mother and risk for adjustment problems in kindergarten: can teachers make a difference? Soc. Dev. 20, 33–50. doi: 10.1111/j.1467-9507.2009.00555.x

[ref10] Calkins S. D. Leerkes E. M. (2011). “Early attachment processes and the development of emotional self-regulation,” in Handbook of Self-Regulation: Research, Theory, and Applications, eds. Vohs K. D. Baumeister R. F.. 2nd ed (New York, NY, United States: Guilford Press), 355–373.

[ref11] Chan S. M. Bowes J. Wyver S. (2009). Parenting style as a context for emotion socialization. Early Educ. Dev. 20, 631–656. doi: 10.1080/10409280802541973

[ref12] Chen X. French D. C. (2008). Children's social competence in cultural context. Annu. Rev. Psychol. 59, 591–616. doi: 10.1146/annurev.psych.59.103006.093606, 18154504

[ref13] Cohen J. (1992). A power primer. Psychol. Bull. 112, 155–159. doi: 10.1037/0033-2909.112.1.155 19565683

[ref14] Curby T. W. Rimm-Kaufman S. E. Ponitz C. C. (2009). Teacher–child interactions and children's achievement trajectories across kindergarten and first grade. J. Educ. Psychol. 101, 912–925. doi: 10.1037/a0016647

[ref15] Denham S. A. Bassett H. H. Wyatt T. (2015). “The socialization of emotional competence,” in Handbook of Socialization: Theory and Research, eds. Grusec J. E. Hastings P. D.. 2nd ed (New York, NY, United States: Guilford Press), 590–613.

[ref16] Eisenberg N. Cumberland A. Spinrad T. L. (1998). Parental socialization of emotion. Psychol. Inq. 9, 241–273. doi: 10.1207/s15327965pli0904_1, 16865170 PMC1513625

[ref17] Eisenberg N. Spinrad T. L. Eggum N. D. (2010). Emotion-related self-regulation and its relation to children's maladjustment. Annu. Rev. Clin. Psychol. 6, 495–525. doi: 10.1146/annurev.clinpsy.121208.131208, 20192797 PMC3018741

[ref18] Enders C. K. (2010). Applied Missing Data Analysis.New York, NY, United States: Guilford Press.

[ref19] Fiorilli C. De Stasio S. Di Chicchio C. Chan S. M. (2015). Emotion socialization practices in Italian and Hong Kong-Chinese mothers. Springerplus 4:758. doi: 10.1186/s40064-015-1550-1, 26682111 PMC4671984

[ref20] Gottman J. M. Katz L. F. Hooven C. (1996). Parental meta-emotion philosophy and the emotional life of families: theoretical models and preliminary data. J. Fam. Psychol. 10, 243–268. doi: 10.1037/0893-3200.10.3.243

[ref21] Gottman J. M. Katz L. F. Hooven C. (1997). Meta-Emotion: How Families Communicate Emotionally. Mahwah, NJ, United States: Lawrence Erlbaum Associates.

[ref22] Graziano P. A. Reavis R. D. Keane S. P. Calkins S. D. (2007). The role of emotion regulation in children's early academic success. J. Sch. Psychol. 45, 3–19. doi: 10.1016/j.jsp.2006.09.002, 21179384 PMC3004175

[ref23] Hajal N. J. Paley B. (2020). Parental emotion and emotion regulation: a critical target of study for research and intervention to promote child emotion socialization. Dev. Psychol. 56, 403–417. doi: 10.1037/dev0000864, 32077713

[ref24] Hamre B. K. Pianta R. C. (2001). Early teacher–child relationships and the trajectory of children's school outcomes through eighth grade. Child Dev. 72, 625–638. doi: 10.1111/1467-8624.00301 11333089

[ref25] Hamre B. K. Pianta R. C. (2005). Can instructional and emotional support in the first-grade classroom make a difference for children at risk of school failure? Child Dev. 76, 949–967. doi: 10.1111/j.1467-8624.2005.00889.x, 16149994

[ref26] Han Z. R. Qian J. Gao M. Dong J. (2015). Emotion socialization mechanisms linking Chinese fathers', mothers', and children's emotion regulation: a moderated mediation model. J. Child Fam. Stud. 24, 3570–3579. doi: 10.1007/s10826-015-0158-y

[ref27] Katz L. F. Maliken A. C. Stettler N. M. (2012). Parental meta-emotion philosophy: a review of research and theoretical framework. Child Dev. Perspect. 6, 417–422. doi: 10.1111/j.1750-8606.2012.00244.x

[ref28] Katz L. F. Windecker-Nelson B. (2004). Parental meta-emotion philosophy in families with conduct-problem children: links with peer relations. J. Abnorm. Child Psychol. 32, 385–398. doi: 10.1023/B:JACP.0000030292.36168.30, 15305544

[ref29] Lagacé-Séguin D. G. Coplan R. J. (2005). Maternal emotional styles and child social adjustment: assessment, correlates, outcomes and goodness of fit in early childhood. Soc. Dev. 14, 613–636. doi: 10.1111/j.1467-9507.2005.00320.x

[ref30] Lunkenheimer E. S. Shields A. M. Cortina K. S. (2007). Parental emotion coaching and dismissing in family interaction. Soc. Dev. 16, 232–248. doi: 10.1111/j.1467-9507.2007.00382.x

[ref31] Mashburn A. J. Pianta R. C. Hamre B. K. Downer J. T. Barbarin O. A. Bryant D. . (2008). Measures of classroom quality in prekindergarten and children's development of academic, language, and social skills. Child Dev. 79, 732–749. doi: 10.1111/j.1467-8624.2008.01154.x, 18489424

[ref32] Morris A. S. Criss M. M. Silk J. S. Houltberg B. J. (2017). The impact of parenting on emotion regulation during childhood and adolescence. Child Dev. Perspect. 11, 233–238. doi: 10.1111/cdep.12238

[ref33] Morris A. S. Silk J. S. Steinberg L. Myers S. S. Robinson L. R. (2007). The role of the family context in the development of emotion regulation. Soc. Dev. 16, 361–388. doi: 10.1111/j.1467-9507.2007.00389.x, 19756175 PMC2743505

[ref34] Mortensen J. A. Barnett M. A. (2018). Emotion regulation, harsh parenting, and teacher sensitivity among socioeconomically disadvantaged toddlers in child care. Early Educ. Dev. 29, 143–160. doi: 10.1080/10409289.2017.1371560

[ref35] Mortensen J. A. Barnett M. A. (2019). Intrusive parenting, teacher sensitivity, and negative emotionality on the development of emotion regulation in Early head start toddlers. Infant Behav. Dev. 55, 10–21. doi: 10.1016/j.infbeh.2019.01.004, 30825714

[ref36] Pianta R. C. La Paro K. M. Hamre B. K. (2008). Classroom Assessment Scoring System (CLASS) Manual, K–3. Baltimore, MD, United States: Paul H. Brookes.

[ref37] Preacher K. J. Curran P. J. Bauer D. J. (2006). Computational tools for probing interactions in multiple linear regression, multilevel modeling, and latent curve analysis. J. Educ. Behav. Stat. 31, 437–448. doi: 10.3102/10769986031004437

[ref38] Qiu C. Shum K. K.-m. (2022). Emotion coaching intervention for Chinese mothers of preschoolers: a randomized controlled trial. Child Psychiatry Hum. Dev. 53, 61–75. doi: 10.1007/s10578-020-01101-6, 33389390

[ref39] Raval V. V. (2023). From the margins to the center: advancing research on caregiver socialization of emotion in Asia. Child Dev. Perspect. 17, 142–148. doi: 10.1111/cdep.12487, 40046247

[ref40] Raver C. C. (2004). Placing emotional self-regulation in sociocultural and socioeconomic contexts. Child Dev. 75, 346–353. doi: 10.1111/j.1467-8624.2004.00676.x 15056189

[ref41] Rogosa D. (1980). A critique of cross-lagged correlation. Psychol. Bull. 88, 245–258. doi: 10.1037/0033-2909.88.2.245

[ref42] Sabol T. J. Pianta R. C. (2012). Recent trends in research on teacher–child relationships. Attach Hum. Dev. 14, 213–231. doi: 10.1080/14616734.2012.672262, 22537521

[ref43] Sankalaite S. Huizinga M. Dewandeleer J. Xu C. de Vries N. Hens E. . (2021). Strengthening executive function and self-regulation through teacher–student interaction in preschool and primary school children: a systematic review. Front. Psychol. 12:718262. doi: 10.3389/fpsyg.2021.718262, 34489822 PMC8417378

[ref44] Shields A. Cicchetti D. (1997). Emotion regulation among school-age children: the development and validation of a new criterion Q-sort scale. Dev. Psychol. 33, 906–916. doi: 10.1037/0012-1649.33.6.906, 9383613

[ref45] Shields A. Cicchetti D. (1998). Reactive aggression among maltreated children: the contributions of attention and emotion dysregulation. J. Clin. Child Psychol. 27, 381–395. doi: 10.1207/s15374424jccp2704_2, 9866075

[ref46] Shortt J. W. Stoolmiller M. Smith-Shine J. N. Mark Eddy J. Sheeber L. (2010). Maternal emotion coaching, adolescent anger regulation, and siblings' externalizing symptoms. J. Child Psychol. Psychiatry 51, 799–808. doi: 10.1111/j.1469-7610.2009.02207.x, 20059622 PMC3601745

[ref47] Society for Research in Child Development (2021). Ethical standards for research with children. Available online at: https://www.srcd.org/about-us/ethical-principles-and-standards-developmental-scientists (Accessed March 10, 2026).

[ref48] Tao A. Zhou Q. Wang Y. (2010). Parental reactions to children's negative emotions: prospective relations to Chinese children's psychological adjustment. J. Fam. Psychol. 24, 135–144. doi: 10.1037/a0018974, 20438189

[ref49] Thompson R. A. (1994). Emotion regulation: a theme in search of definition. Monogr. Soc. Res. Child Dev. 59, 25–52. doi: 10.1111/j.1540-5834.1994.tb01276.x 7984164

[ref50] Trevethan M. Lin K. L. Raval V. V. Li X. Hu J. Deo N. (2021). Mothers' emotion socialization profiles and relation to adolescent socio-emotional functioning in China and India. J. Appl. Dev. Psychol. 73:101259. doi: 10.1016/j.appdev.2021.101259

[ref51] Verschueren K. Koomen H. M. Y. (2012). Teacher–child relationships from an attachment perspective. Attach Hum. Dev. 14, 205–211. doi: 10.1080/14616734.2012.672260, 22537520

[ref52] Wang M. Liang Y. Zhou N. Zou H. (2019). Chinese fathers' emotion socialization profiles and adolescents' emotion regulation. Personal. Individ. Differ. 137, 33–38. doi: 10.1016/j.paid.2018.08.006

[ref53] Zelazo P. D. Carlson S. M. (2012). Hot and cool executive function in childhood and adolescence: development and plasticity. Child Dev. Perspect. 6, 354–360. doi: 10.1111/j.1750-8606.2012.00246.x

